# Pervasive heteroplasmy in an invasive ambrosia beetle (Scolytinae) in southern California

**DOI:** 10.1038/s41437-024-00722-0

**Published:** 2024-09-12

**Authors:** Paul F. Rugman-Jones, Christine E. Dodge, Richard Stouthamer

**Affiliations:** 1 Department of Entomology, University of California, Riverside, CA 92521 USA; 2 Present Address: Forest Pest Methods Laboratory, USDA-APHIS-PPQ-S&T, 1398 W. Truck Rd, Buzzards Bay, MA 02542 USA

**Keywords:** Evolutionary theory, Haplotypes, Invasive species, Genetic techniques

## Abstract

Heteroplasmy, the presence of multiple mitochondrial genotypes (mitotypes) within an individual, has long been thought to be a rare aberrance that is quickly removed by selection or drift. However, heteroplasmy is being reported in natural populations of eukaryotes with increasing frequency, in part due to improved diagnostic methods. Here, we report a seemingly stable heteroplasmic state in California populations of the polyphagous shothole borer (PSHB), *Euwallacea fornicatus*; an invasive ambrosia beetle that is causing significant tree dieback. We develop and validate a qPCR assay utilizing locked nucleic acid probes to detect different mitotypes, and qualitatively assess heteroplasmy in individual PSHB. We prove the utility of this assay by: (1) mitotyping field-collected PSHB, documenting the prevalence of heteroplasmy across its range in California; and, (2) measuring relative titers of each mitotype across multiple generations of heteroplasmic laboratory colonies to assess the stability of transmission through the maternal germline. We show that our findings are unlikely to be explained by the existence of NUMTs by next generation sequencing of contiguous sections of mitochondrial DNA, where each of the observed heteroplasmic sites are found within fully functional coding regions of mtDNA. Subsequently, we find heteroplasmic individuals are common in Californian field populations, and that heteroplasmy persists for at least 10 generations in experimental colonies. We also looked for evidence of the common occurrence of paternal leakage, but found none. In light of our results, we discuss competing hypotheses as to how heteroplasmy may have arisen, and continues to perpetuate, in Californian PSHB populations.

## Introduction

Among the scientific community, the long-held consensus belief is that the vast majority of sexually reproducing organisms inherit their mitochondria from only one parent, their mother. It has been proposed that this uniparental inheritance evolved to suppress the spread of selfish and/or deleterious mitochondrial DNA (mtDNA) variants that may impose fitness costs on a cell or organism (Felsenstein 1974; Law and Hutson 1992; Bergstrom and Pritchard 1998; Hadjivasiliou et al. 2012; Chen et al. 2020). By extension, it is accepted that mtDNA must also be inherited strictly through the maternal line, and be self-replicating, which results in a state of homoplasmy; all cells of an individual organism harbor identical copies of the mtDNA.

In contrast, mitochondrial heteroplasmy occurs when multiple distinct copies of the mtDNA are present in the same individual. Heteroplasmy must occur naturally since mutations in existing mtDNA are required to produce new mitochondrial genotypes (mitotypes), but it is traditionally thought to be a transient state, as genetic bottlenecks and selection act to quickly return organisms to being homoplasmic for either the original, or the new mitotype (Ladoukakis and Zouros 2017; Parakatselaki and Ladoukakis 2021). However, a growing number of studies of natural populations of eukaryotic species, have revealed instances where heteroplasmy is seemingly a common, and often stable, state (Kmiec et al. 2006; Doublet et al. 2008; Davies et al. 2022; Francoso et al. 2023). Among these studies, arthropods are especially well-represented (Rand and Harrison 1986; Boyce et al. 1989; Nardi et al. 2001; Van Leeuwen et al. 2008; Magnacca and Brown 2010; Nunes et al. 2013; Wolff et al. 2013; Chandler et al. 2015; Robison et al. 2015; Gawande et al. 2017; Kastally and Mardulyn 2017; Meza-Lazaro et al. 2018; Sriboonlert and Wonnapinij 2019; Davies et al. 2022; Francoso et al. 2023).

In addition to the acquisition of natural sequence mutations, heteroplasmy can also result if there is a breakdown in the mechanisms that effectively ensure that paternal mitochondria (or mtDNA) are excluded from fertilized ova, or if such mechanisms are simply not as robust and/or ubiquitous as assumed; a phenomenon commonly referred to as paternal leakage (reviewed by, Schwartz 2021). Among arthropods, paternal leakage has been described in only a handful of species, including fruit flies *Drosophila* spp. (Nunes et al. 2013; Polovina et al. 2020), 17-year periodical cicadas *Magicicada* spp. (Fontaine et al. 2007) and brown dog ticks *Rhipicephalus* spp. (Mastrantonio et al. 2019). In all three groups, hybrid mating between heterospecific congeners occasionally resulted in the production of heteroplasmic offspring, replete with mitochondria from both parents. Although instances of conspecific paternal leakage have also been reported in insects (e.g., several species of *Drosophila*; Wolf et al. 2013; Nunes et al. 2013; Sherengul et al. 2006; Polovina et al. 2020), it has been suggested that paternal leakage should be a more common occurrence following mating between heterospecific crosses due to reduced efficiency in the egg’s recognition of heterospecific sperm mitochondria (Rokas et al. 2003; Dokianakis and Ladoukakis 2014). In short, if some factor coded in the maternal nuclear genome fails to recognize a divergent “signal” coded on the outer surface of sperm mitochondria, the elimination of those mitochondria may not be triggered (Ladoukakis and Zouros 2017).

Within an organism, heteroplasmy may manifest itself at several, not mutually exclusive, levels (Phillips et al. 2015; Rollins et al. 2016). Heteroplasmy may result from intercellular variation (different cells harbor distinct mitotypes) or intracellular variation (a single cell harbors multiple distinct mitotypes). It may also result from variation at the molecular level since mitochondria typically harbor multiple copies of their mtDNA (e.g., Robin and Wong 1988), one or more of which may differ. Such variation at the molecular level might be considered “pseudo”-heteroplasmy if each one of an organism’s mitochondria harbors the very same molecular variation (i.e. the mitochondrion itself is heteroplasmic, but the individual is essentially homoplasmic). Regardless of the level at which variation occurs, orthologous copies of mtDNA may differ in their DNA sequences, and/or their length, and it is the widespread adoption of DNA sequencing that has doubtless driven the discovery of heteroplasmy in a growing number of invertebrate species.

Unfortunately, DNA sequencing also has the potential to create a fallacy. The phenomena described above all assume that only orthologous copies of mtDNA are being sequenced. However, the integration and recombination of fragments of mtDNA into the nuclear genome, referred to as Nuclear Mitochondrial DNA (and abbreviated as NUMT; Lopez et al. 1994), is increasingly recognized as a common occurrence in eukaryotic organisms (Zhang and Hewitt 1996; Bensasson et al. 2001; Song et al. 2008; Gaziev and Shaikhaev 2010). Once inserted into the nuclear genome, NUMT sequences may acquire mutations independent of their mitochondrial source DNA, which could in turn manifest as the appearance of heteroplasmy if the two paralogous stretches of DNA are incidentally amplified in the same PCR reaction (Song et al. 2008). A recent extensive study focusing on the cytochrome c oxidase I (COI) gene region of mtDNA of 1002 insect species, detected at least one NUMT in around 84% of those species (Hebert et al. 2023). However, across orders, genome size was found to be a positive determinant of the occurrence of NUMTs, and NUMTs were relatively rare in the four most speciose orders, Coleoptera, Diptera, Hymenoptera, and Lepidoptera, which typically have small genomes (Hebert et al. 2023). Furthermore, the majority of NUMTs identified in this study were found to possess indels and/or premature stop codons, allowing their easy recognition. That said, this was by no means always the case, with some NUMTS harboring only one or two synonymous substitutions, and clearly, vigilance should be afforded in the examination of any apparent case of heteroplasmy.

The polyphagous shothole borer (PSHB) *Euwallacea fornicatus* (Eichhoff) (Coleoptera: Curculionidae: Scolytinae) is a highly pestiferous ambrosia beetle, in the tribe Xyleborini. The Xyleborini is highly diverse, and probably the most ecologically and economically significant group of ambrosia beetles, due to the penchant of several of its members for attacking healthy trees, as opposed to the dead or dying trees favored by most ambrosia beetles (Hulcr and Dunn 2011). PSHB is native to South East Asia, but in recent years has established invasive populations in several areas around the world, including the U.S. (California and Hawaii), Israel, South Africa, and Australia (Rabaglia et al. 2006; Mendel et al. 2012; Stouthamer et al. 2017; Paap et al. 2018; Rugman-Jones et al. 2020; Pest and Disease Information Service 2021). Like other ambrosia beetles, PSHB is dependent for its food, on symbiotic fungi that it carries along. The beetles burrow into trees creating galleries in the xylem tissues, the walls of which are seeded with spores of the symbiotic fungi. The fungi extract nutrients from the plant, and the beetles feed exclusively on the fungi. However, in the case of PSHB, the ambrosial fungi are also phytopathogenic to a large number of tree species (Eskalen et al. 2013; UCANR 2023). The result is the plant disease Fusarium dieback, which, in invaded areas, has led to significantly increased mortality of trees in natural, urban, and agricultural landscapes (Eskalen et al. 2013; Boland 2016; de Wit et al. 2022; Cook and Broughton 2023). Like other Xyleborini, PSHB is a haplodiploid species, with flightless haploid males, and female-biased populations that inbreed almost exclusively (Gadd 1941, 1947; Kirkendall 1993; Normark et al. 1999). Females typically mate with a brother within their natal gallery prior to dispersal, but even in the event that a female is not inseminated before leaving the natal gallery, she can also potentially mate with a haploid son produced from an unfertilized egg (Cooperband et al. 2016). Such arrhenotokous sex determination paired with sibling-mating essentially produces something akin to a clonal lineage (Andersen et al. 2012), which in turn reduces genetic variation within populations. This reduction in genetic variation is likely to be even more profound in areas where these beetles are recently invasive due to bottlenecks and other founder effects. Such mating systems have received little attention, but it has been theorized that heavily inbred species may have relaxed controls on mitochondrial inheritance, perhaps promoting heteroplasmy (Hurst 1994).

Based on a 658 bp fragment of the COI “barcoding” gene, two distinct mitotypes of PSHB have been identified in California, which differ from each other at only a single nucleotide position; hereafter referred to as H33 and H35 following the haplotype assignations given by Stouthamer et al. (2017). As part of on-going efforts to map the geographic distribution and spread of these two invasive mitotypes (plus a single haplotype of a second, congeneric and morphologically identical, invasive species, the Kuroshio shothole borer, *Euwallacea kuroshio* Gomez and Hulcr), a diagnostic assay based on high-resolution melt analysis (HRM) of a short fragment of the COI gene was initially designed to rapidly identify *Euwallacea* specimens based on their mitotype (Rugman-Jones and Stouthamer 2017). However, whilst mitotyping beetles using this HRM assay, we encountered several PSHB specimens that displayed a distinctive, but erroneous, “shouldered” melt curve (Fig. S1a). Subsequent Sanger sequencing of the COI region of these specimens (as per Stouthamer et al. 2017) suggested they were in fact heteroplasmic, with the presence of both the H33 and H35 mitotypes within each individual, as evidenced by a double peak at the polymorphic site in the sequence chromatograms (Fig. S1b).

Here we report the incidence of heteroplasmy in different PSHB populations across Southern California. We designed a locked nucleic acid (LNA) probe-based qPCR assay that allowed us to accurately identify heteroplasmic individuals and infer the relative contribution of the H33 and H35 mitotypes in such individuals. We subsequently use that assay to map the geographic distribution of heteroplasmic PSHB in the invasive Californian population, and explore the trans-generational stability of heteroplasmy across multiple generations of lab-maintained colonies reared with different development rates. Furthermore, to conclusively rule out the possibility that this heteroplasmy might be due to the occurrence of a NUMT, we assemble the mitochondrial genome of beetles of differing plasmic status (H33, H35, and heteroplasmic). In light of our findings, we examine competing hypotheses for how heteroplasmy may have arisen in the California population.

## Methods

### Development and validation of a locked nucleic acid (LNA)-probe based assay for the relative quantitation of mitotype titers

The sequences of a 657 bp fragment of the barcoding region of the COI gene of haplotypes H33 and H35 (GenBank accessions JX912724 and JX912723, respectively) differ by only a synonymous single nucleotide polymorphism (SNP) at position 598 (Eskalen et al. 2013; Table S1). A qPCR-based assay, incorporating multiplexed LNA probes targeting this SNP site, was designed by Integrated DNA Technologies, Inc. (IDT; Coralville, IA, USA) (Table 1). An LNA, often referred to as inaccessible RNA, is a modified RNA nucleotide in which the ribose moiety is modified with an extra bridge connecting the 2’ oxygen and 4’ carbons. When incorporated into a probe, these LNA bases can increase its hybridization performance, greatly improving mismatch discrimination (i.e. SNP genotyping) compared to a classical dual-labeled (e.g. TaqMan) probe (Johnson et al. 2004; You et al. 2006; Josefsen et al. 2009). Primers for a 92 bp fragment, and a differentially labeled LNA probe for each mitotype (Table 1) were synthesized by IDT, supplied in a lyophilized format, re-eluted in ultrapure water to a concentration of 10 μM, and divided into “single-use” aliquots in opaque amber-colored microcentrifuge tubes to minimize any potential effects of repeated freeze-thawing or exposure to light. Aliquots were stored at −20 °C until use in the qPCR assay.

**Table 1:** PCR primers and LNA probes for relative quantification of mitotype titers.

Primer/Probe	Oligo sequence (5′–3′)	Bases	*T*_m_ (°C)^a^
PSHB-HetCOI-F	TAGCTGGAGGAATCACCATATT	22	61.0
PSHB-HetCOI-R	TTGATATAGGACAGGATCTCCAC	23	61.0
H33-Probe^b^	HEX-TG + A + G + GTGT + T + A + ATATTTC-IABkFQ^c^	17	63.8
H35-Probe^b^	FAM-AG + G + T + GT + T + G + ATATT-IABkFQ^c^	13	64.1

Primers and PrimeTime® LNA® probes were designed and synthesized by Integrated DNA Technologies, Inc.

^a^Information supplied by manufacturer.

^b^Nucleotides preceded by a “+” indicate an incorporated LNA monomer.

^c^Probe quencher IABkFQ = Iowa Black FQ®.

LNA probes are typically used for diagnostic (e.g., genotyping) purposes and not absolute quantitation. However, we were interested in whether two differently-labeled probes could be combined in a single reaction to estimate the relative titers of the two mitotypes (H33 and H35) in heteroplasmic individuals. This could be used to quantify changes in titers over time, to see if one mitotype increased in frequency as the other decreased in frequency; in other words, if we could observe a trend toward homoplasmy. In order to test this, we first extracted DNA from each of 10 individuals known to be either H33 or H35 (i.e. non-heteroplasmic). For this, we performed simple DNA extractions in which the entire specimen was incubated in 150 μl TE buffer (10 mM Tris-Cl, 0.5 mM EDTA; pH 9.0) for 30 min at 95 °C. The DNA of each mitotype was pooled resulting in ~1.5 mL each of H33 and H35 DNA. We assumed that these were of approximately equal concentration: although not formerly quantified, we have no reason to believe that individuals of one mitotype would have more mitochondria (and therefore yield more mitochondrial DNA) than individuals of the other. The pooled DNA extractions were then used to make a series of mixes in the following ratios (H33:H35); 90:10, 80:20, 70:30, 60:40, 50:50, 40:60, 30:70, 20:80, and 10:90. These mixes were used to test the ability of the LNA-probe assay to qualitatively discriminate different relative titers.

The LNA-probe assay was conducted in 20 μL reactions, each containing 1x HOT FIREPol® Probe qPCR Mix Plus (no ROX) (Mango Biotechnology, Mountain View, CA, USA), 250 nM each primer, 100 nM each probe (Table 1), and 2 μL template DNA. Four replicate reactions were run for each mix, plus the two “pure” haplotypes and a negative control in which TE buffer was added instead of DNA. The assay was conducted on a Rotor-Gene Q or Rotor-Gene 3000 thermocycler (Qiagen), using a 2-step profile with the following settings: 95 °C for 15 min (to activate the HOT FIREPol® polymerase), followed by 40 cycles of 95 °C for 10 s and 60 °C for 30 s, with acquisition of fluorescence data (on the yellow [H33] and green [H35] channels) at the end of each 60 °C step. On completion, fluorescence data were exported to Excel (Microsoft) and for each channel, two measures were “extracted” for each individual sample. The first measure, “baseline” (or background) fluorescence, was calculated as the mean fluorescence reading at the end of each cycle from 3–15; a period during which there was no detectable amplification (window of linearity). A second, “endpoint” fluorescence reading was extracted at the end of the exponential phase of the qPCR by calculating the mean fluorescence reading at the end of each cycle from 31–35. Amplification success was quantified as change in fluorescence (*df*), calculated by subtracting the baseline from the endpoint fluorescence. The four replicates were then used to calculate mean *df*. Since the yellow and green channels measure fluorescence on different scales, the two were normalized to a single scale by rescaling *df* of each sample based on the *df* of the negative and positive controls using the formulae:

$${\rm{Normalized}}\,{df}[{\rm{yellow}}]=\frac{({df}[{\rm{yellow}}]X-{df}[{\rm{yellow}}]{\rm{NTC}})}{({df}[{\rm{yellow}}]{\rm{H}}33-{df}[{\rm{yellow}}]{\rm{NTC}})}$$

and,

$${\rm{Normalized}}\,{df}[{\rm{green}}]=\frac{({df}[{\rm{green}}]X-{df}[{\rm{green}}]{\rm{NTC}})}{({df}[{\rm{green}}]{\rm{H}}35-{df}[{\rm{green}}]{\rm{NTC}})}$$

where *X* is the sample, H33 and H35 are pure (positive) controls, and NTC is the TE buffer (negative) control. The rescaled data for each sample were then plotted in a 100% stacked column chart, to show the relative quantification of H33:H35 for each sample/specimen. The entire validation process (from making mixes to rescaling the fluorescence data) was repeated three times. On the third occasion, the range of mix ratios was extended to include 95:5 and 5:95 (H33:H35).

**Table 2:** Incidence of heteroplasmy across five field populations of PSHB in Southern California: La Habra Heights (LHH); Irvine Regional Park, Orange (IRP); Prado Dam and Santa Ana River, Corona (COR); the Huntington Library, Art Collections, and Botanical Gardens, Pasadena (PAS); and, various sites in Ventura County (VEN).

		Mitotype *N* (%)		
Locality	*N*^a^	H33	H35	Heteroplasmic
LHH	100	53 (53.0)	39 (39.0)	8 (8.0)
IRP	104	19 (18.3)	83 (79.8)	2 (1.9)
COR	143	30 (21.0)	102 (71.3)	11 (7.7)
PAS	102	15 (14.7)	69 (67.6)	18 (17.6)
VEN	107	0 (0.0)	69 (64.5)	38 (35.5)

The mitotype of each individual specimen was determined using the LNA-probe assay.

^a^Total number of PSHB typed.

### Heteroplasmy or NUMT?

To rule out the existence of a NUMT we followed two different approaches. We first looked for evidence of heteroplasmy in mature eggs from heteroplasmic mothers. Mature eggs, prior to the onset of embryogenesis, are expected to contain a very high number of mitochondria relative to the number of copies of the nuclear genome (Woods et al. 2018). Thus, our ability to co-amplify any NUMT (i.e. to detect “heteroplasmy”) should be greatly reduced, although, as embryogenesis proceeds, we might expect the number of mitochondria relative to the number of copies of the nuclear genome to ease towards a more harmonious balance (i.e. any NUMT would again become more obvious). Eighteen mature eggs were collected from the brood galleries of a heteroplasmic laboratory colony maintained at 26 °C. Each individual egg was predicted to be anywhere between 1 and 7 days old, but since PSHB females rarely lay more than a single egg per day, it was highly unlikely that the eggs were all the same age (Gadd 1947). To extract DNA, individual eggs were homogenized in 1 μL of proteinase-K and 50 μL of a 5% suspension of Chelex100 resin, and incubated for 1 h at 56 °C. Extractions were terminated with 10 min at 99 °C, and centrifuged for 4 min at 14,000 rpm to pellet the resin. Two microliters of the supernatant were subsequently used as template in the LNA-probe assay (see above). Using this method, DNA was also extracted from a single egg collected from a homoplasmic H33 and H35 lab colony to act as controls.

The second approach was to determine the complete mitochondrial genome of the beetles using a next generation sequencing approach. A single ethanol-preserved female beetle collected from a laboratory colony of each mitotype (H33, H35, and heteroplasmic) was surface sterilized by gentle shaking in a 2% sodium hyperchlorite solution for 2 min. The specimens were then rinsed with ultrapure water and whole genomic DNA was extracted only from the legs of each specimen using a Nucleospin® DNA Insect Kit (Macherey Nagel GmbH and Co. KG, Dűren, Germany). This process aimed to minimize any external contamination and remove the possibility of co-extracting DNA from stored sperm of an unrelated male. The legs were “harvested” under a dissecting microscope and transferred to a NucleoSpin® Bead Tube Type D containing 100 μl Buffer BE and 40 μl Buffer MG. Samples were homogenized, for 1 min at 3500 rpm, in a BeadBug™ 3 benchtop homogenizer (Benchmark Scientific, Sayreville, NY, USA) and then centrifuged for 30 s at 11,000 × *g*. To each homogenized sample we then added 10 μl Liquid Proteinase K. The samples were vortexed briefly to mix and then incubated at 56 °C for 1 h to complete lysis. DNA was bound to a Nucleospin® DNA Insect Column and washed following the manufacturer’s protocol and the final elution of the DNA was done using 60 μl of Elution Buffer BE, which was passed through the column twice to maximize yield. The extractions were subsequently ethanol precipitated and re-eluted in 25 μl ultrapure water and the DNA was quantitated using a Qubit^TM^ 2.0 (Life Technologies, Waltham, MA, USA) with the Qubit^TM^ dsDNA HS Assay Kit. Total DNA yield for the three samples averaged 43.1 ± 13.6 ng. The NEBNext Microbiome DNA Enrichment Kit (New England Biolabs, Ipswich, MA, USA) was then used to increase the ratio of mitochondrial to nuclear DNA in each extract. While this kit is designed to facilitate enrichment of microbial DNA from samples containing methylated host DNA, it may also be effective for separation of organellar DNA from eukaryotic nuclear DNA (Yigit et al. 2014). Enrichment was performed according to the manufacturer’s protocol with ethanol precipitation as the final purification step, the DNA being resuspended in 20 μl of ultrapure water.

Illumina (San Diego, CA, USA) libraries, with an average insert size of 200 bp, were prepared by the Institute of Integrative Genome Biology, University of California, Riverside, and sequenced using the Illumina NovaSeq S4 platform at the Vincent J. Coates Genomics Sequencing Laboratory (QB3 Genomics, UC Berkeley, Berkeley, CA, RRID:SCR_022170) with 150 bp paired-end reads. The combined data yield for the three libraries totaled almost 13 Gb, constituting 71.8 million raw reads. Prior to assembly, the reads were trimmed to remove the Illumina adapters, ambiguous base calls (maximum number of ambiguities = 2), and poor quality reads (quality score limit = 0.05), using CLC Genomic Workbench version 6.0.2. The trimmed reads were subsequently assembled into contigs, using the de novo assembly function in CLC Genomics Workbench, with a minimum contig length of 1000, and otherwise default de novo options. The option to map reads back to the contigs (again with default options) was also selected. The resulting contigs were examined for evidence of the DNA sequence of the target locus for our LNA assay (Table 1) using BLASTn with the “Align two or more sequences” function, enabling the retrieval of a more-or-less complete mitochondrial genome for each mitotype, and ensuring that the locus was not repeated in any of the remaining contigs. The mitochondrial contigs were examined for ambiguities (conflicts) using the “Extract Consensus Sequence” function in CLC Genomics Workbench with a low coverage threshold of 20x and a noise threshold of 0.05 (i.e. any conflict must be present in at least 5% of the mapped reads). Having identified any ambiguous sites, the three mitochondrial genomes were transferred to BioEdit and aligned with an existing *E. fornicatus* mitochondrial genome (MT897842) retrieved from GenBank. Following alignment, the conflicting/ambiguous positions in one mitochondrial genome were subsequently reassessed across all three mitochondrial genomes for evidence of lower levels of conflict (<5% disagreement), in CLC Genomics Workbench.

### Incidence of heteroplasmy in field populations

The prevalence of heteroplasmy in southern California PSHB populations was assessed using specimens garnered from monitoring trap samples collected between 2014 and 2019. At least 100 alcohol-preserved specimens from each of five localities across southern California were mitotyped: La Habra Heights (LHH); the Huntington Library, Art Collections, and Botanical Gardens, Pasadena (PAS); Prado Dam and Santa Ana River, Corona (COR); Irvine Regional Park, Orange (IRP); and various sites in Ventura County (VEN). Simple DNA extraction in TE buffer was performed as described earlier, and 2 μl of the resultant DNA template was used to assay each specimen using the LNA-probe assay (as described above). Plasmic state (H33, H35, or heteroplasmic) was determined based on the fluorescent signal from the two LNA “reporter” probes.

The historical incidence of heteroplasmy in southern California was also assessed. Stouthamer et al. (2017) included two pinned “museum” specimens that were collected in the Whittier Narrows area of Los Angeles County in 2003, around the time that PSHB was first detected in California (Rabaglia et al. 2006). Both specimens were identified by Stouthamer et al. (2017) as the H35 mitotype, but seven further pinned specimens, collected in the same area around the same time (2003–2004), were also extracted but could not be sequenced (Rugman-Jones, pers. com.). DNA degradation is a common problem in pinned specimens and this additional material did not yield DNA of sufficient quality to allow amplification and sequencing of the 657 bp fragment of COI utilized by Stouthamer and colleagues (2017). However, since our LNA probe assay relies on the amplification of a fragment of only 92 bp, we “revisited” these extractions/specimens using the LNA probe assay.

### Trans-generational stability of mitochondrial heteroplasmy

Heteroplasmy has generally been considered a transient state that is rapidly eliminated by selection or drift (Aanen et al. 2014). For example, in heteroplasmic *Drosophila* spp., it has been shown that the frequency of one particular mitotype (L) consistently increases in frequency between mothers and their offspring, and furthermore, the size of this increase grows bigger as a mother ages (Solignac et al. 1987; Kann et al. 1998; Rand 2011). We investigated the transmission of heteroplasmic mtDNA through the female germ line, by comparing the frequency of the H33 and H35 mitotypes in individual beetles drawn from successive generations of heteroplasmic PSHB. Replicate laboratory colonies were initiated on artificial diet media (Carrillo et al. 2020) using field-caught heteroplasmic females. Each generation, over the course of several days, female adult offspring were isolated as they emerged from the natal gallery and either genotyped (to assess heteroplasmy) or transferred to new diet media to initiate the next generation. We also manipulated the temperature at which the colonies were maintained (23 °C vs. 29 °C) to determine how development time might affect transmission (see Rand 2011); PSHB reared at 23 °C develop much slower than at 29 °C (Umeda and Paine 2018). Three separate colonies were maintained at each temperature for approximately one year, during which, the average relative frequency of the two mitotypes was measured using the LNA-probe assay. Samples of individual offspring (*n* ⋝ 4) were assayed at generations 1 and 6 (both temperature regimes), and also at the 10th generation for the colonies maintained at 29 °C.

### Is heteroplasmy a common outcome following hybridization between H33 and H35?

In most organisms, it is typically thought that cellular mechanisms are in place that effectively ensure that paternal mitochondria (or mtDNA) are excluded from fertilized ova (i.e. preventing paternal leakage), thereby maintaining homoplasmy. However, we hypothesized that such mechanisms may be less important in a mating system like that of the Xyleborini, where females typically mate with their brothers (Normark et al. 1999), and both parents therefore harbor identical mitochondria. Under this hypothesis, occasional outbreeding events may foster paternal leakage, perpetually creating heteroplasmic offspring. To test this, we set up crosses between H33 and H35 individuals. PSHB were isolated by destructively sampling several homoplasmic H33 and H35 laboratory colonies. To ensure their virginity, and to produce enough males to use in crosses, females from each of three lines per haplotype were collected as pupae, and maintained on water agar plates. After eclosing, individual females were transferred to plates of *Fusarium euwallaceae*, the primary fungal symbiont of PSHB (Freeman et al. 2013), and allowed to feed for 24 h in order to populate their mycangia with the fungal spores necessary to feed their future broods. Males for these crosses were collected from the broods of virgin foundresses that were allowed to establish colonies on artificial diet media and, without access to sperm, could only produce haploid male offspring. Individual males and virgin females were subsequently paired to create two ‘hybrid’ crosses (H33 female x H35 male, and H35 female x H33 male), and the paired beetles were placed in 50 mL centrifuge tubes containing artificial diet media and maintained at 26 °C for 5 weeks. This time period was chosen to allow enough time for the F_1_ offspring to begin completing their development, while ensuring there was not enough time for an F_2_ generation. Under this scenario, only females that mated with their original counterpart (and subsequently fertilized their eggs) would have enough time to produce daughters. Female F_1_ offspring were collected as they emerged and euthanized in 95% ethanol. Subsequently, five offspring were chosen at random from each of 18 broods, and the plasmic status of each was determined using the LNA-probe assay.

## Results

### Validation of the locked nucleic acid (LNA)-probe assay

**Fig. 1:**
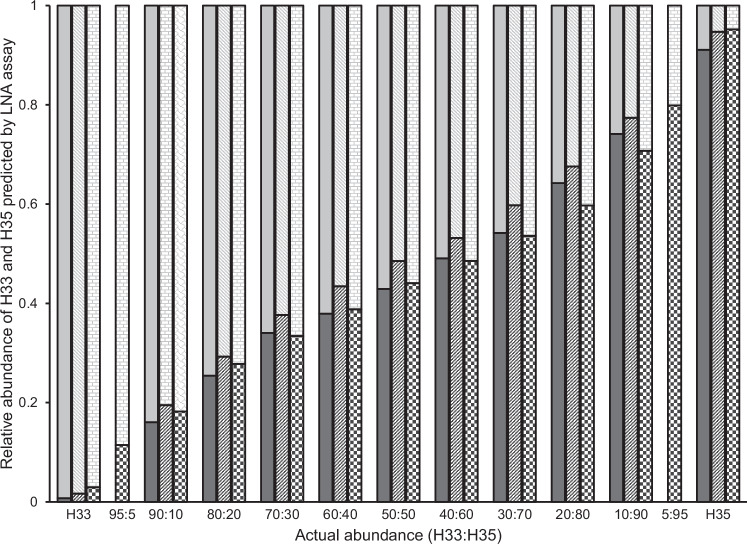
The ability of an LNA probe assay to measure heteroplasmy in PSHB. Incremental mixes of H33 and H35 were prepared from pooled homoplasmic DNA (H33+ and H35+; *x*-axis). The abundance of the H33 (light gray) and H35 (dark gray) mitotypes relative to each other was then estimated in each mix based on the fluorescence emitted by their respective LNA probes following normalization to a single scale (see text). Validation was performed in three replicate runs (solid bars vs. striped bars vs. patterned bars, respectively). The third run was extended to include the two mitotypes in a ratio of 95:5 relative to each other. No-template controls were also run but are not plotted since their incorporation into the normalization of the change in fluorescence of each probe results in zero values.

The LNA-probe assay accurately documented heteroplasmy and, at least qualitatively, was able to reflect changes in the relative abundance of the two haplotypes, H33 and H35 (Fig. 1). The H33 probe was slightly more prone to cross-hybridization (i.e. binding to the wrong allele) than the H35 probe, which can be seen by comparing their relative levels of fluorescence in the alternative homologous control (i.e. H33+ or H35+), but in neither case was cross-hybridization sufficient to interfere with the ability of the assay to detect heteroplasmy even when one haplotype was 19-fold more abundant than the other (Fig. 1, replicate 3).

### Heteroplasmy or NUMT?

**Fig. 2:**
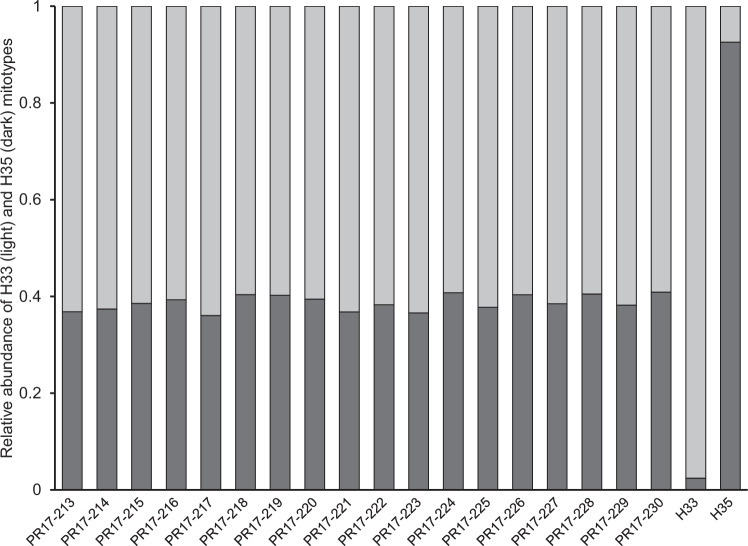
The abundance of H33 (light gray) and H35 (dark gray) mitotypes relative to each other in mature eggs collected from a heteroplasmic PSHB colony. PR numbers represent individual eggs. Rightmost two columns represent control DNA extracted from eggs collected from laboratory-maintained homoplasmic H33 and H35 colonies.

We hypothesized that if the apparent occurrence of heteroplasmy was in fact due to the co-amplification of a copy of the COI that had become inserted in the nuclear genome (NUMT), then the high abundance of mitochondria in mature eggs (relative to the low number of nuclear copies), would minimize the chances of such co-amplification. Across 18 mature eggs collected from a heteroplasmic laboratory colony, the relative titer of H33:H35 was around 40:60 and remarkably uniform (Fig. 2). Given that the age of those eggs was almost certainly not uniform, such a pattern would not be expected if a NUMT was involved. This strongly suggests that we were observing genuine variation in mitochondrial DNA and not some kind of pseudo-heteroplasmy as a result of co-amplification of a NUMT.

To add further weight to this conclusion, we assembled more-or-less complete mitochondrial genomes for a single beetle of each mitotype (H33, H35, and heteroplasmic). A single contig of approximately 15 Kb, and with >99% similarity to an existing *E. fornicatus* mitochondrial genome (MT897842) was extracted from 150 bp PE Illumina data for each mitotype (Table S2). Average depth of coverage was over 660 for each contig, and as expected of a mitochondrial genome, there was a distinct AT bias in its DNA sequence (~72.5%; Table S2). The heteroplasmic position identified in COI and targeted by our LNA probe assay was confirmed as ambiguous in the heteroplasmic beetle (40% cytosine and 60% thiamine at a coverage depth of x531), but was completely homoplasmic in each of the “pure” mitotypes (100% thiamine at a coverage depth of x482 in H33, and 100% cytosine at a depth of x620 in H35; Table S2). The remainder of the three mitogenomes (outside the COI fragment) were identical with the exception that, a second ambiguous position, was identified in the H33 contig. At a coverage depth of x999, a position in NADH dehydrogenase subunit 1 was found to be 49% thiamine and 51% cytosine, but in both the H35 and heteroplasmic beetle, at a similar coverage depth (x1027 and x997, respectively), the position was more-or-less 100% cytosine (with two reads in each library, attributed to adenosine: Table S2).

### Incidence of heteroplasmy in field populations

**Fig. 3:**
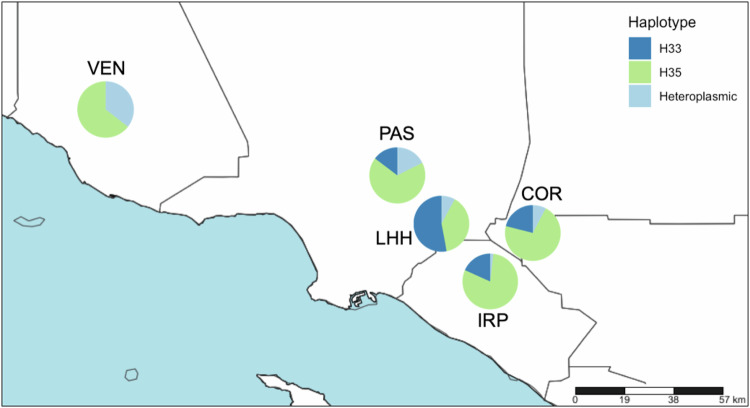
Haplotype composition of PSHB populations sampled in five areas of Southern California. Lines represent county borders, and blue shading designates the Pacific Ocean. Exact values for percentages and numbers of specimens are listed in Table 2.

A total of 556 individuals were assayed from monitoring trap samples of five localities in southern California. Following only a very simple DNA extraction (boiling the specimen in TE buffer), 100% of the individuals yielded a result. Thus, the LNA probe assay found evidence that heteroplasmic individuals were present in all locations sampled (Fig. 3 and Table 2). At four of the five localities, H35 was the dominant haplotype (Fig. 3). The exception was around the La Habra Heights area, where H33 dominated. There was significant geographic variation in the occurrence of heteroplasmy with the percentage of beetles within a population that were heteroplasmic ranging from 1.9% (IRP) to 35.5% (VEN). Perhaps surprisingly, we found that only H35 and heteroplasmic PSHB were present in samples from Ventura County (Fig. 3 and Table 2). At the intra-individual level, it was rare to find heteroplasmic specimens in which the relative abundance of one mitotype to the other exceeded a 3-fold difference (Fig. S2).

**Fig. 4:**
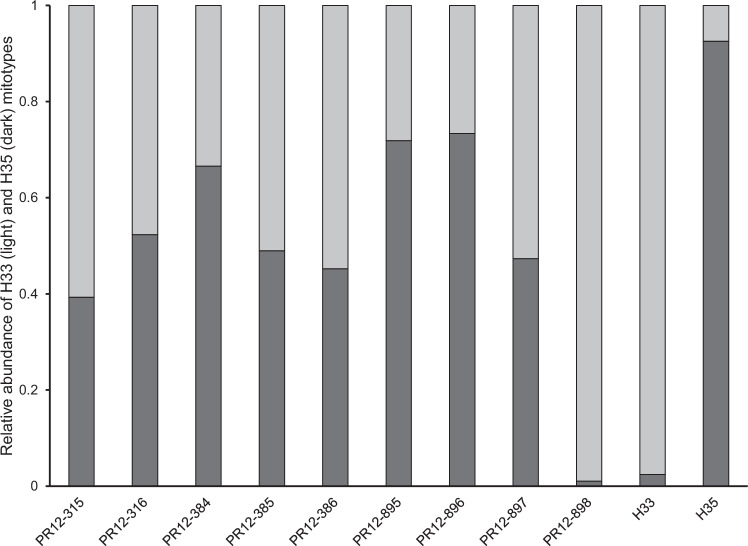
Historical abundance of heteroplasmy in the PSHB population in Los Angeles County, California. The abundance of the H33 (light gray) and H35 (dark gray) mitotypes relative to each other was estimated using an LNA probe assay (see text). PR numbers represent individual specimens that were collected in 2003–2004 and subsequently dried and pinned. PR12-315, -316, -384, -385, and -386 collected in Avocado Heights, 10-IX-2004; PR12-895 and -896 were collected in Whittier Narrows Rec. Area, 30-V-2003 and 30-X-2003, respectively and were identified as H35 in Stouthamer et al. (2017); PR12-897 was collected in Whittier Narrows Rec. Area, 30-XI-2003; PR12-898 was collected in Whittier, 6-IX-2004. Rightmost two columns represent control DNA extracted from homoplasmic H33 and H35 beetles.

The LNA assay was also successful at typing the nine pinned specimens dating from 2003–2004. One specimen was identified as H33 but the others all appeared to be heteroplasmic, including the two specimens (PR12-895 and PR12-896) from Stouthamer et al. (2017) that were previously identified as H35 (Fig. 4). In both of those specimens, the relative titer of the two mitotypes was heavily skewed toward H35. Thus, 8 of 9 specimens that were collected around the time (and locality) that PSHB was first detected in California, were found to be heteroplasmic.

### Trans-generational stability of heteroplasmy

**Fig. 5:**
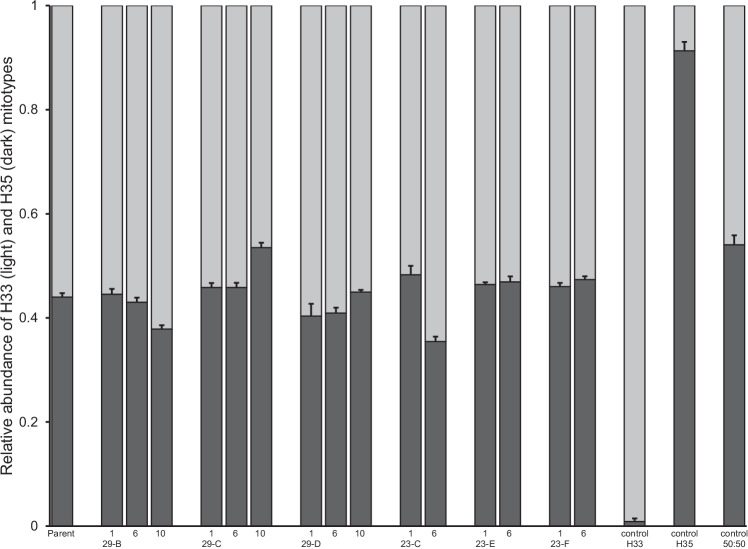
Relative mitotype titers in six iso-female lineages of PSHB instigated from a heteroplasmic parental colony (P) and reared at 29 °C (29-B, -C, and -D) or 23 °C (23-C, -E, and -F). Titers were estimated for each lineage from a minimum of four individuals at generations 1 and 6 (all lineages), and in lineages reared at 29 °C, also at generation 10. The abundance of H33 (light gray) and H35 (dark gray) mitotypes relative to each other was estimated using an LNA probe assay (see text). Homoplasmic H33 and H35 DNA stocks, and a 50:50 mix of the two, were used as positive controls. No-template controls were also run but are not plotted since their incorporation into the normalization of the change in fluorescence of each LNA probe results in zero values.

Over the course of 6 or 10 generations, respectively, heteroplasmic colonies of PSHB reared at either 23 °C or 29 °C, were subject to only small changes in the relative titers of the H33 and H35 mitotypes (Fig. 5). In addition, there was no apparent directional selection for either mitotype, under either of the temperature regimes.

### Is heteroplasmy a common outcome following hybridization between H33 and H35?

**Fig. 6:**
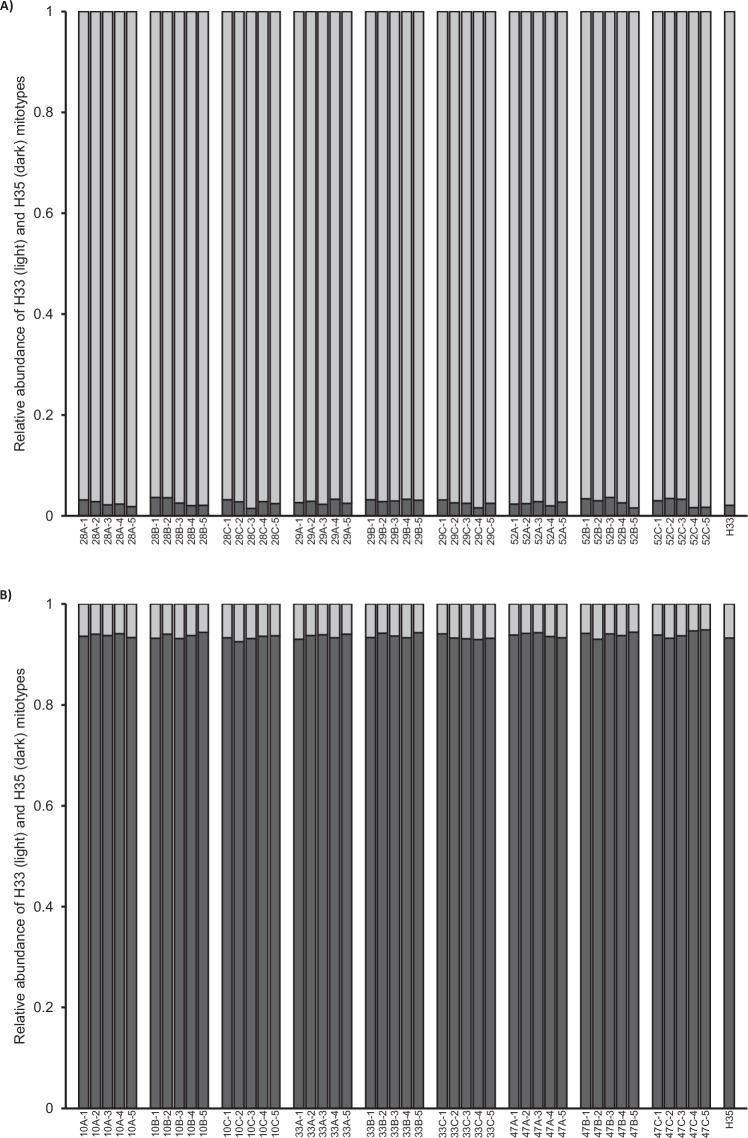
No evidence of paternal leakage in crosses between individuals with differing mitotypes. Relative mitotype titers in the “hybrid” female offspring of: **A** H33 mothers mated to an H35 male; and **B** H35 mothers mated to an H33 male. Three “replicate” females were selected from each of three laboratory colonies of each homoplasmic mitotype and paired with a male of the other mitotype and reared on artificial diet. Daughters resulting from the pairings were collected as they emerged. Subsequently, five daughters were randomly selected from each brood and typed using the LNA-probe assay. Rightmost column is a homoplasmic control.

No evidence was found for the production of heteroplasmic female offspring following mating between females or males from an H33 colony and a partner drawn from an H35 colony. All daughters tested were found to harbor only their mother’s mitotype (Fig. 6).

## Discussion

The widespread adoption of DNA sequencing, and derived molecular methods, has contributed to revealing the occurrence of heteroplasmy in a growing number of invertebrate species. However, heteroplasmy is still considered a rare and transient condition that typically occurs at low frequencies, both within individuals and within populations. Contrary to these expectations, this study found that heteroplasmy in Californian populations of PSHB, an invasive ambrosia beetle, appears to be both common and stable. With the aid of a newly developed LNA probe assay (Fig. 1), across southern California, heteroplasmic individuals were found in each of five surveyed populations at frequencies up to 35% (Fig. 3 and Table 2), and within those heteroplasmic individuals, the relative contribution of two distinct mitotypes (H33:H35) was found to be typically in the range 75:25 to 25:75 (Fig. S3). Temperature (and by extension, development rate) did not appear to effect transmission of heteroplasmy through the female germline, and, at least over 10 generations in the laboratory, relative titers of H33 and H35 showed no clear shift towards homoplasmy (Fig. 5). We confirmed that this heteroplasmy was not likely an erroneous phenomenon brought about by the existence of a NUMT in two ways. First, by showing that it remained detectable in eggs, where mtDNA is expected to be much more abundant than nuclear DNA (Fig. 2), and subsequently, by sequencing more-or-less the entire mitochondrial genome of beetles thought to be H33, H35, or heteroplasmic (Table S2). We also showed that heteroplasmy was maternally transmitted and that its apparent stability was not due to the perpetual (re)creation of heteroplasmic offspring due to paternal leakage in crosses involving parents with “pure” but opposing mitotypes (Fig. 6). While the period over which our laboratory crosses were monitored (10 generations) may not have been long enough for drift or purifying selection to show a clear effect, further support for the stable nature of heteroplasmy in Californian PSHB was provided by the observation that heteroplasmic individuals were present in field populations as far back as 2003 (Fig. 4), when PSHB was first detected in California, and have therefore persisted for at least two decades.

### LNA probe assay

The LNA probe assay presented herein proved a reliable quantitative and qualitative measure for detecting heteroplasmy and assessing mitotype titers in invasive Californian populations of PSHB, even in pinned museum specimens with low quality DNA. Furthermore, it worked well with pinned museum specimens, allowing us to track the existence of heteroplasmic PSHB individuals in California back to when it was first detected in 2003. Many of those early specimens had been rejected in earlier work (Stouthamer et al. 2017) due to failing to amplify a bigger COI target, presumably due to DNA degradation. We have also used the assay subsequently to confirm that all beetles from Israel and Vietnam, identified as H33 by Stouthamer et al. (2017), were homoplasmic (data not shown).

### Origin and maintenance of heteroplasmy in Californian populations of PSHB

The present study provides strong evidence that heteroplasmy in Californian populations of PSHB is real, common, and stable. Based on these findings, we propose two competing hypotheses for the origins of heteroplasmic PSHB. The first is that the heteroplasmy represents an ongoing transitional stage that has resulted in the “founding” of the H35 mitotype. All discrete mitotypes must start out as a mutation in the mitochondria of a single individual, and therefore exist for a period in a heteroplasmic state. Over time, the new mitotype may replace (or be replaced by) the original mitotype due either to some selective advantage, or simply to drift. While in addition to California, H33 has been recovered from populations of PSHB in its native range in Southeast Asia, and from other invasive populations in Israel and South Africa, the H35 mitotype has not been detected anywhere else outside of California (Stouthamer et al. 2017). Thus, it is possible that, prior to or soon after the establishment of PSHB in California, a mutation in the H33 mitotype occurred during oogenesis, giving rise to a new mitotype, H35, and the first heteroplasmic mother. That H35 was created as a result of some “point mutation” in H33 is further supported by our sequence of the whole mitogenome of a beetle with each mitotype, which revealed that the nucleotide difference initially detected in a 658 bp fragment of the COI gene was in fact the only difference between the two mitotypes across the mitogenome (~15,000 bp).

The second hypothesis is that the heteroplasmy resulted from paternal leakage following mating between H33 and H35 individuals. Paternal leakage occurs when mitochondria are inherited through the paternal line as well as the maternal line (Schwartz 2021). It has been suggested that it should be a more common occurrence following mating between genetically distant parents (e.g., heterospecific crosses) due to reduced efficiency in the egg’s recognition of sperm mitochondria; some factor coded in the maternal nuclear genome fails to recognize a divergent “signal” coded on the outer surface of sperm mitochondria, and the elimination of those mitochondria is not triggered (Ladoukakis and Zouros 2017). However, the chances of paternal leakage occurring between closely related (homospecific) forms may be increased in highly inbred taxa such as xyleborine beetles (Hurst 1994). In this group, females typically mate with their brothers (Normark et al. 1999), and as a result, mothers and fathers are expected to harbor identical mitochondria. In such an inbred mating-system, cellular mechanisms that typically exclude a sperm’s mitochondria from the egg pre- or post-fertilization (see Sato and Sato 2013; but also see Schwartz 2021) may be under weakened selection (Hurst 1994), perhaps because the sperm’s mitochondria are “expected” to be identical to those found in the egg. Thus, following a chance outbreeding event between H33 and H35 individuals, male mitochondria may escape elimination, resulting in heteroplasmic offspring. This only had to happen once, but could also be reasonably common if non-sib mating in fact occurs more frequently in PSHB than has previously been thought for other Xyleborines (Normark et al. 1999). We tested this under laboratory conditions but were unable to “recreate” such paternal leakage in reciprocal crosses between H33 and H35 parents. Coupled with a lack of any evidence for heteroplasmic individuals in native populations (e.g., none were detected among those sequenced by Stouthamer et al. 2017), this suggests that paternal leakage is indeed rare.

Whether heteroplasmy in southern California populations of PSHB arose following some mutation that made it through the oogenic mitochondrial bottleneck, or as a result of some rare instance of paternal leakage, following its genesis, the heteroplasmic state itself could be deleterious, neutral, or advantageous to individuals. If heteroplasmy is deleterious, we should expect to see a rapid return to homoplasmy in the population. The fact that heteroplasmy can be vertically transmitted over multiple generations (10) in laboratory colonies, and continues to persist in a fraction of the southern Californian population of PSHB (20 years and counting) suggests that it is at least not strongly selected against in this species. If heteroplasmy is effectively neutral, this would support the idea that the Californian population of PSHB is still undergoing some drawn out period of “purification”, via drift or weak selection, which will eventually return the population to a homoplasmic state. Although in mammals, it has been shown that homoplasmy can be restored very rapidly (2–3 generations; Ashley et al. 1989), studies of other insects have estimated that several hundred generations may be required before heteroplasmy is completely removed from a population (Solignac et al. 1984; Rand and Harrison 1986). Thus, the current state of heteroplasmy in the Californian population of PSHB can be viewed as transient and the population will eventually return to a homoplasmic state, composed of a matrix of H33 and H35 individuals. The fact that eight of nine specimens drawn from some of the earliest collections of PSHB in California were found to be heteroplasmic (Fig. 4), and that homoplasmic individuals now dominate, suggests that such purification may indeed be occurring, albeit slowly.

However, we have thus far ignored the possibility that heteroplasmy may in fact convey some kind of advantage to the beetles. The two mitotypes may provide offsetting advantages under changing environmental conditions, and under such a scenario, it has been suggested that mitochondrial heteroplasmy could be favored by mechanisms akin to balancing selection and heterozygote advantage, that act on nuclear genomes (Davies et al. 2022). In an expanding invasion, heteroplasmic populations may subsequently adapt to new environments, since heteroplasmy could potentially facilitate selection on competing mitotypes (Rollins et al. 2016). Interestingly, H35 was the most abundant mitotype at all but one of our field sampling sites, the exception being La Habra Heights (LHH; Fig. 2 and Table 2). The location of LHH is very close to that of the earliest (2003) California collections of PSHB in Whittier Narrows, and the other sites are known to have been colonized some time later. If some germline mutation in the founding population gave rise to H35, and heteroplasmic individuals, perhaps H35 is now slowly coming to prominence as the invasion expands, because of some selective advantage it has over H33 in Southern California. Although this is a tempting explanation, in truth, drawing any meaningful inferences about the fitness of H33, H35, or heteroplasmic PSHB, or the continued persistence of heteroplasmic individuals in the field, will require a resampling of field populations in future years.

Finally, our study shines little light on the actual source of the heteroplasmic “signal” in our PSHB populations. Essentially, heteroplasmy can result from variation at various levels (Phillips et al. 2015; Rollins et al. 2016); the cellular level (different host cells contain different mitochondria i.e., with different sequences), the mitochondrion level (different mitochondria within a single host cell have different sequences), and/or the molecular level (each mitochondrion has 2–10 copies of its DNA, which may differ). The extent of variation seen in the relative titers of H33 to H35 among heteroplasmic individuals in field populations (Fig. S3) suggests that, in this instance, heteroplasmy cannot be explained by some kind of “fixed” variation at the molecular level (e.g., each mitochondrion contains a fixed number of copies of the H33 mtDNA and a fixed number of copies of the H35 mtDNA). However, concerted evolution (in different directions) or recombination between mitochondria could lead to inter-individual, molecular level variation in the relative titers of H33 to H35 (Schwartz 2021). Perhaps a more parsimonious explanation though, is that heteroplasmy is the result of inter-cellular or inter-mitochondrial variation, which may more readily lead to such “shifts” in the relative titer of H33 to H35 across individuals. It is also possible that variation occurs at all three levels, and random oppositional drift at each level incidentally fosters the maintenance of heteroplasmy. We have also not considered the full potential extent of DNA sequence variation in the mitochondrial genome of Californian populations of PSHB. Like many of the existing insect studies, our assessment of variation and, therefore, determination of heteroplasmy, is based largely on a short fragment of a single gene (COI), which accounts for only around 4% of the total mitochondrial genome (assuming a typical size of 16 Kb). We sequenced the whole mitogenome of only three individuals, one from each of our H33, H35, and heteroplasmic colonies in order to rule out the possibility of a NUMT. In doing that work, we identified one additional ambiguous nucleotide in the NADH dehydrogenase subunit 1 gene of the H33 beetle, but not in the H35 or heteroplasmic beetles. This discovery suggests that additional heteroplasmic variants, outside the scope of the present study, may be present. Thus, future work might seek to assess the whole mitochondrial genome of a much bigger sample of field-collected PSHB. Continued, temporal sampling of the invasive populations may also help shine a light on the adaptation of this exotic ambrosia beetle to novel habitats in California.

## Supplementary information

Supplementary information

## Data Availability

Fluorescence data from the LNA-probe assays have been deposited in DRYAD 10.5061/dryad.w0vt4b924. Unique COI haplotype data, on which the LNA-probe assay was developed, are available in GenBank (JX912723-JX912724) and the mitogenomes assembled herein have been deposited in the same repository (PQ300101-PQ300103). Benefits from this research accrue from the sharing of our data and results on public databases as described above.
