# Supplementary material for: Pervasive heteroplasmy in an invasive ambrosia beetle (Scolytinae) in southern California
Source: Heredity (Edinb). 2024 Sep 12;133(6):388–99. doi: 10.1038/s41437-024-00722-0 (PMC11589772; doi:10.1038/s41437-024-00722-0)
Supplement: Supplementary file 1 — Supplementary information [file 41437_2024_722_MOESM1_ESM.pdf]

Table S1. Polyphagous shothole borer mitotypes found in southern California; 658bp "barcoding" fragment of 5' COI gene. Shaded area indicates the fragment amplified by the LNA assay.

```
Mitotype                   10         20         30         40         50         60
                  ....|....| ....|....| ....|....| ....|....| ....|....| ....|....|
H33 [JX912724]  AACTCTATAT TTCATTTTTG GTGCATGAGC AGGAATAGTA GGAACATCCC TTAGAGTTCT
H35 [JX912723]  .......... .......... .......... .......... .......... ..........

                           70         80         90        100        110        120
                  ....|....| ....|....| ....|....| ....|....| ....|....| ....|....|
H33 [JX912724]  AATTCGAACC GAATTAGGAA CACCTGGAAG ACTAATTATA GATGATCAAA TTTTTAATAC
H35 [JX912723]  .......... .......... .......... .......... .......... ..........

                          130        140        150        160        170        180
                  ....|....| ....|....| ....|....| ....|....| ....|....| ....|....|
H33 [JX912724]  TATTGTAACA GCCCATGCTT TTATTATAAT TTTCTTTATA GTTATACCTA TTCTAATTGG
H35 [JX912723]  .......... .......... .......... .......... .......... ..........

                          190        200        210        220        230        240
                  ....|....| ....|....| ....|....| ....|....| ....|....| ....|....|
H33 [JX912724]  TGGATTTGGA AACTGGTTAG TTCCATTAAT ACTAGGAGCC CCAGATATAG CATTCCCTCG
H35 [JX912723]  .......... .......... .......... .......... .......... ..........

                          250        260        270        280        290        300
                  ....|....| ....|....| ....|....| ....|....| ....|....| ....|....|
H33 [JX912724]  GCTTAATAAT ATAAGATTTT GGTTATTGCC ACCAAGATTA TCTCTTCTTT TGATAAGAAG
H35 [JX912723]  .......... .......... .......... .......... .......... ..........

                          310        320        330        340        350        360
                  ....|....| ....|....| ....|....| ....|....| ....|....| ....|....|
H33 [JX912724]  AATTATCGAC AAAGGAGCAG GAACAGGATG AACTGTTTAT CCTCCTCTAG CAGCAAATAT
H35 [JX912723]  .......... .......... .......... .......... .......... ..........

                          370        380        390        400        410        420
                  ....|....| ....|....| ....|....| ....|....| ....|....| ....|....|
H33 [JX912724]  CGCCCATGAA GGAGCTTCAA TTGACCTAGC AATTTTTAGA CTTCATATAT CAGGTGTATC
H35 [JX912723]  .......... .......... .......... .......... .......... ..........

                          430        440        450        460        470        480
                  ....|....| ....|....| ....|....| ....|....| ....|....| ....|....|
H33 [JX912724]  ATCAATTCTG GGAGCCATTA ATTTCATCTC AACTATTATT AATATACATC CAAAAGGAAT
H35 [JX912723]  .......... .......... .......... .......... .......... ..........

                          490        500        510        520        530        540
                  ....|....| ....|....| ....|....| ....|....| ....|....| ....|....|
H33 [JX912724]  AAAACCTGAA CAACTAACCT TATTTACTTG AGCAGTAAAA ATCACTGCAA TCTTACTATT
H35 [JX912723]  .......... .......... .......... .......... .......... ..........

                          550        560        570        580        590        600
                  ....|....| ....|....| ....|....| ....|....| ....|....| ....|....|
H33 [JX912724]  ATTGTCTCTA CCCGTACTAG CTGGAGGAAT CACCATATTA CTTACAGATC GAAATATTAA
H35 [JX912723]  .......... .......... .......... .......... .......... .......C..

                          610        620        630        640        650
                  ....|....| ....|....| ....|....| ....|....| ....|....| ....|..
H33 [JX912724]  CACCTCATTT TTTGATCCAG CAGGAGGTGG AGATCCTGTC CTATATCAAC ATCTATT
H35 [JX912723]  .......... .......... .......... .......... .......... .......
```

Table S2. Summary statistics for the sequencing, assembly, and alignment of the mitogenome of H33, H35, and heteroplasmic *Euwallacea fornicatus* (polyphagous shothole borer). Illumina (San Diego, CA, USA) libraries, enriched for mitochondrial DNA using the NEBNext Microbiome DNA Enrichment Kit (New England Biolabs, Ipswich, MA, USA), were prepared by the Institute of Integrative Genome Biology, UCR, and sequenced using the Illumina NovaSeq S4 platform at the Vincent J. Coates Genomics Sequencing Laboratory (QB3 Genomics, UC Berkeley, Berkeley, CA, RRID:SCR_022170) with 150 bp paired-end reads. Contigs (3rd column) were assembled *de novo* in CLC Genomic Workbench version 6.0.2. A single mitochondrial contig was extracted from each library and aligned with an existing *E. fornicatus* mitogenome (GenBank MT897842).

Mitotype	No. of reads after trimming	Total no. of assembled contigs	Assembled mitochondrial contig			Position* 1,970		Position* 11,705	
			Length (bp)	Ave. coverage	A/T content	Coverage	C/T frequency (count)	Coverage	C/T frequency (count)
H33	28,299,796	4,063	15,557	x 661.82	0.724	x 482	0.00/1.00 (0/482)	x 999	0.49/0.51 (493/505)
H35	22,366,201	4,731	14,918	x 877.32	0.728	x 620	1.00/0.00 (620/0)	x 1027	1.00/0.00 (1025/0)§
Heteroplasmic	21,142,950	1,197	14,945	x 777.28	0.728	x 531	0.40/0.60 (212/319)	x 997	1.00/0.00 (995/0)§

*position based on GenBank accession MT897842 (Wang et al., 2020)
§Adenosine at this postion x2

A)

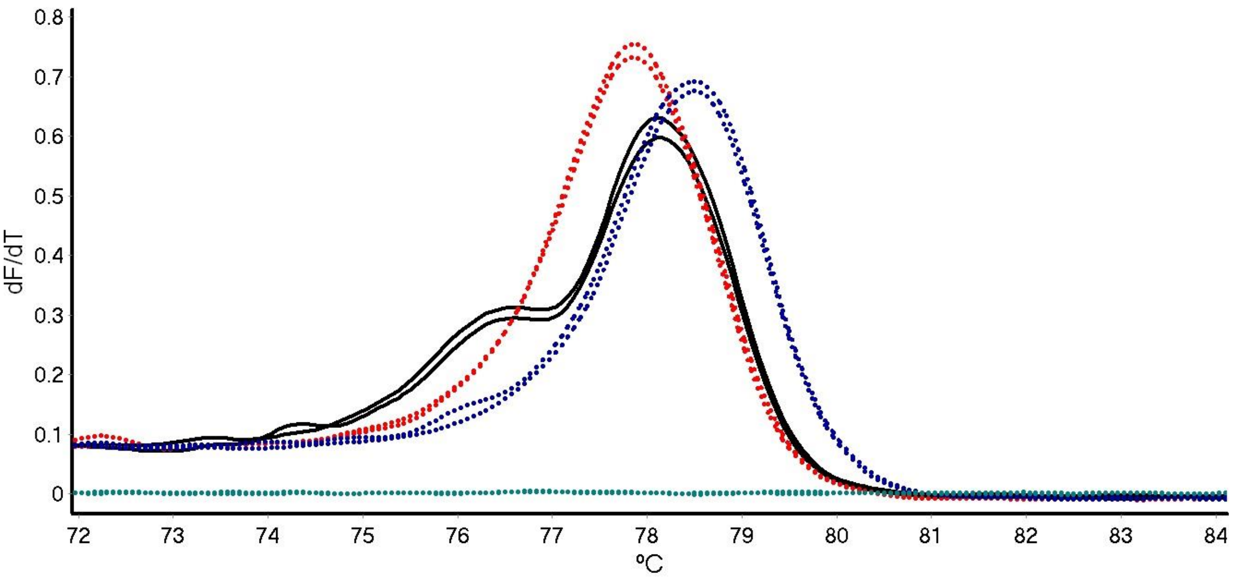

B)

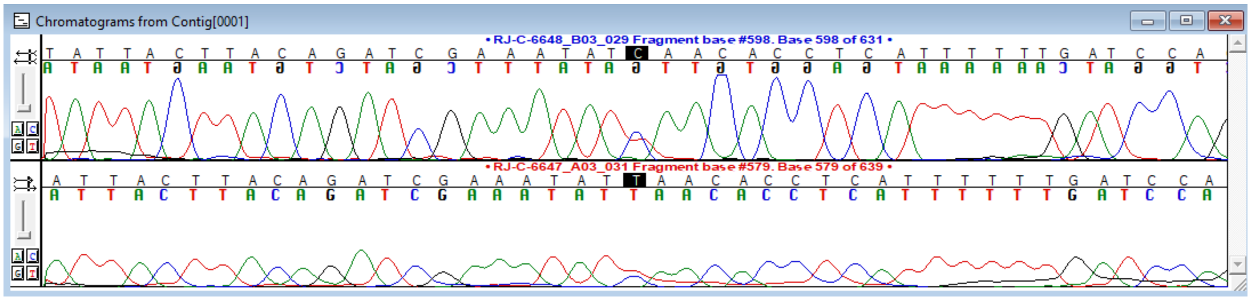

Figure S1. Heteroplasmy in southern California specimens of PSHB. Distinctive melt curves (A) produced by an 84bp PCR-amplified fragment of the COI gene (see Rugman-Jones & Stouthamer 2016). Red and blue dotted lines represent individuals harboring H33 or H35 haplotypes, respectively; black-solid lines represent individuals that were subsequently confirmed as heteroplasmic (i.e. harboring both H33 and H35) by a double peak in Sanger sequencing traces (B).

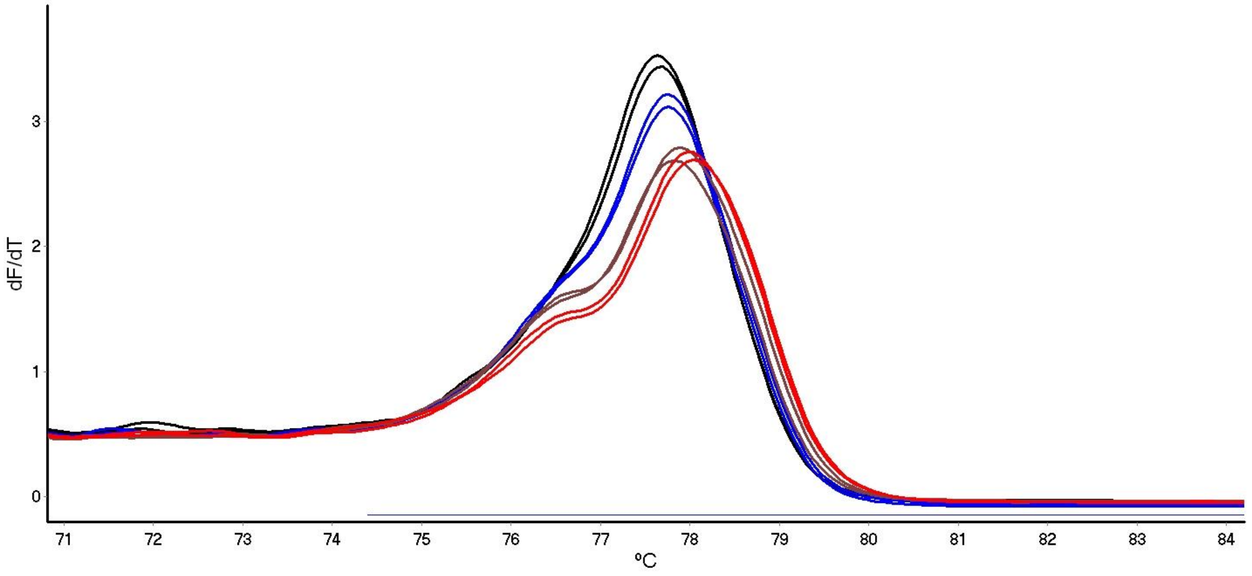

Figure S2. High-resolution melt analysis is an inaccurate method for recording heteroplasmy in southern California populations of PSHB. Heteroplasmic DNA was created artificially by mixing H33 and H35 DNA in four different ratios. Heteroplasmy was first detected following the production of a melt curve with a distinct shoulder (see Fig. S1), but in heteroplasmic mixes where H33 exceeds H35 (black lines = 80:20, blue lines = 60:40), that shoulder was found to be much less pronounced compared to the reciprocal mixes (brown lines = 40:60, red lines = 20:80) which could result in the mis-identifcation of heteroplasmic individuals (in which H33 is the dominant mitotype) as homoplasmic H33.

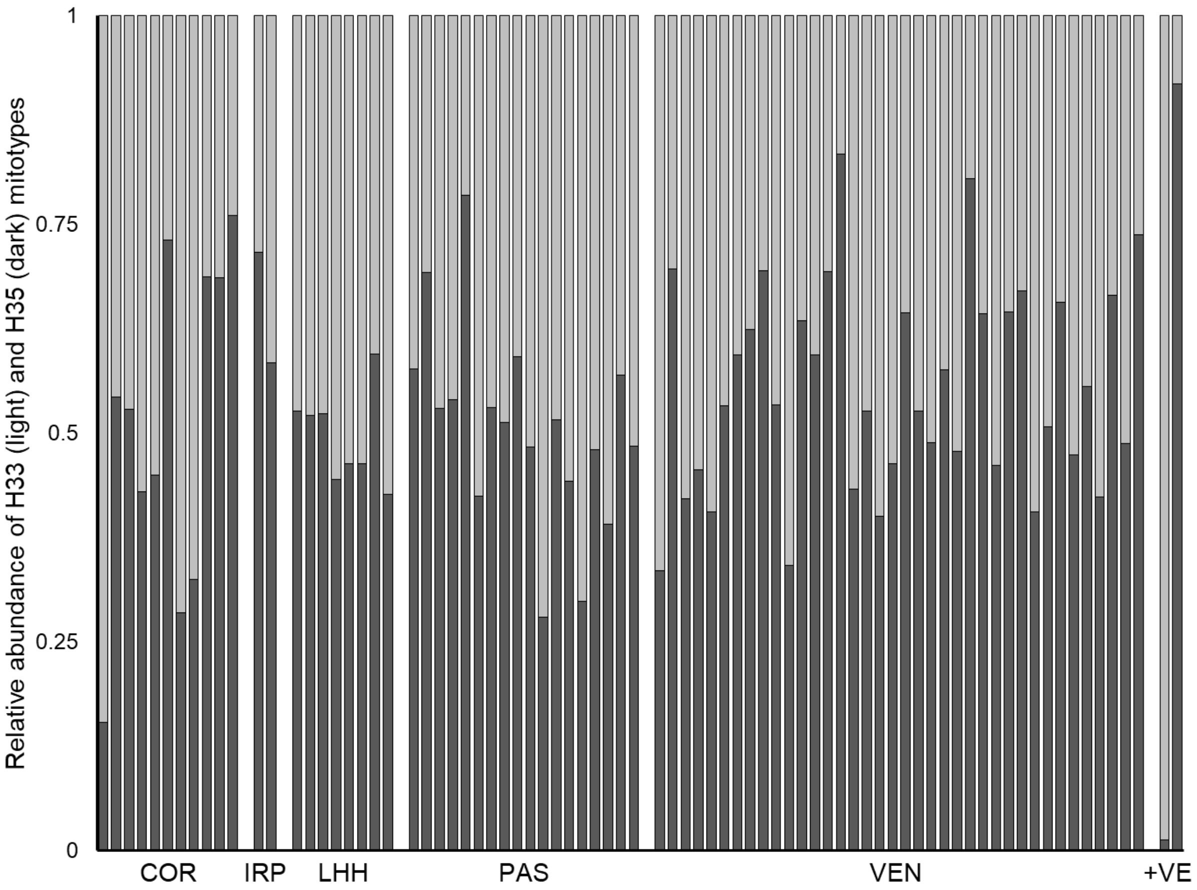

Figure S3. Relative titer of two mitotypes, H33 (light grey) and H35 (dark grey) in heteroplasmic individuals from five field populations of PSHB in southern California: Prado Dam and Santa Ana River, Corona (COR); Irvine Regional Park, Orange (IRP); La Habra Heights (LHH); the Huntington Library, Art Collections, and Botanical Gardens, Pasadena (PAS); and, various sites in Ventura County (VEN). Rightmost two columns represent control DNA extracted from homoplasmic H33 and H35 beetles.
